# A Cross-Sectional Study of the Dietary Carbon Footprints of US Schoolchildren

**DOI:** 10.3390/nu18101529

**Published:** 2026-05-12

**Authors:** Andrea Barney, Donald Rose, Amelia Willits-Smith, Lori Andersen Spruance

**Affiliations:** 1 Department of Nutrition, Dietetics, and Food Science, Brigham Young University, S221 ESC, 701 E University Parkway, Provo, UT 84602, USA; anbarney@gmail.com; 2 Department of Social, Behavioral, and Population Sciences, Cecilia Scott Weatherhead School of Public Health and Tropical Medicine, Tulane University, 1440 Canal Street, New Orleans, LA 70112, USA; diego@tulane.edu (D.R.);; 3 Department of Public Health, Brigham Young University, 4103 LSB, 701 E University Parkway, Provo, UT 84602, USA

**Keywords:** carbon footprint, dietary quality, children, adolescents, climate impact

## Abstract

**Objectives**: This study’s main objective was to examine the association between dietary greenhouse gas emissions (GHGEs) and diet quality among U.S. primary and secondary schoolchildren. Our secondary objectives were to identify demographic characteristics associated with higher-GHGE diets and to assess the relationship between school meal consumption and dietary GHGE. **Methods**: We conducted a cross-sectional secondary analysis of dietary intake data from 2165 primary and secondary students participating in the nationally representative 2014–2015 US School Nutrition and Meal Cost Study (SNMCS). Dietary GHGEs (kg CO_2_-equivalents per 1000 kcal) were estimated by linking reported foods to an environmental impact database. Diet quality was assessed using the Healthy Eating Index-2010 (HEI). Students were categorized into GHGE groups, and differences in total HEI and component scores were examined using appropriate statistical tests. Statistical significance was set at *p* < 0.05. **Results**: The average dietary GHGE of the sample was 3.64 kg CO_2_-equivalent per person per day. Overall, HEI scores did not differ between the GHGE groups (*p* = 0.22). However, compared to the high-GHGE group, the low-GHGE group scored significantly higher on some HEI-2010 components, such as fatty acid ratios (*p* < 0.0001) and sodium (*p* < 0.0001), and significantly lower on others, such as dairy (*p* < 0.0001), total protein foods (*p* < 0.0001), and refined grains (*p* < 0.0001). Male students and students who ate school meals on the day of recall had higher odds of being in the high-GHGE group compared with their respective reference groups. **Conclusions**: Lower dietary carbon footprints are achievable without sacrificing overall diet quality, but tradeoffs in specific dietary components indicate the need for additional research and care in recommending diet changes or setting school policies.

## 1. Introduction

Global climate change is a pressing issue with well-documented environmental and health impacts. Recent reports indicate record high global temperatures, ocean heat content, and declining snow and sea ice extent [1], alongside growing evidence linking climate change to adverse human health outcomes [2].

The food sector is a significant contributor to climate change, accounting for about a third of greenhouse gas emissions (GHGEs) [3]. Over half of the emissions come from the production of livestock and other animal-based foods, which significantly contribute to methane and nitrous oxide emissions [4]. Animal-based foods are typically associated with a larger environmental impact compared to plant-based foods [5,6]. Since food production is shaped by consumer demand, there is an urgent need to align human diets with the goals of environmental sustainability [7,8]. Plant-based dietary patterns are generally associated with benefits for both environmental health and human health [9,10]. Plant-based foods, such as fruits and vegetables, contain bioactive compounds that can reduce oxidative stress and inflammation, thereby improving cardiovascular, gut, and brain health [10]. Higher intake of whole grains, fruits and vegetables, legumes, and nuts has been associated with reduced risk of cardiovascular disease and type 2 diabetes [11,12]. Changing eating habits to include more plant-based foods and fewer animal-based foods could have lasting effects on both human and environmental health [9,12]. Studies have examined the effects of high meat intakes on health and have found relationships between processed and red meat consumption and colorectal cancer [13], cardiovascular disease [14], and diabetes [15].

Dietary habits formed during childhood and adolescence typically carry on into adulthood [16,17,18]. While diet quality scores have increased slightly over time, there is still room for improvement given that the estimated proportion of youth in the United States with poor diets was almost 40% in 2015–2016 [19]. Finding opportunities to improve eating habits could improve both health and the environment [9,12]. However, the relationship between dietary quality and environmental impact is complex and not uniformly aligned [19]. Diets with lower greenhouse gas emissions (GHGEs) are often characterized by reduced consumption of animal-source foods and greater intake of plant-based foods, which may align with dietary recommendations emphasizing fruits, vegetables, and whole grains [19]. At the same time, important trade-offs exist: Some nutrient-dense foods, such as dairy and certain animal-source products, contribute disproportionately to GHGEs while providing essential nutrients [5,20]. These patterns reflect underlying mechanisms linking diet to both outcomes, including differences in the environmental impacts of the production of different foods and differences in their nutritional composition. Together, these dynamics underscore the importance of examining dietary patterns through both nutritional and environmental lenses to better understand potential synergies and trade-offs.

Other countries have examined the GHGE of children’s diets. In the Netherlands, researchers estimated that the average GHGE for a day’s consumption is 3.2 kg of CO_2_-equivalents for girls aged 7 to 18 and 3.6 kg of CO_2_-equivalents for boys aged 7 to 18 [20]. Temme et al. also found that intakes of total fat, saturated fat, and animal-based protein were significantly higher in the high-GHGE group compared to the low-GHGE group, and there was also lower fiber intake [21]. A study of Swedish adolescents found that dietary greenhouse gas emissions ranged from 3.8 to 4.2 kg CO_2_-equivalents [22]. This study determined that a higher Healthy Eating Index score was associated with lower GHGEs [22]. To our knowledge, the dietary greenhouse gas emissions of US children and their relationship with healthy eating indices or food groups have not yet been explored in a national sample.

Several sociodemographic factors are associated with high-GHGE diets. Understanding these associations is important for developing possible interventions to reduce dietary GHGEs. Lindroos et al. also found that males were likely to have higher dietary GHGEs compared to females in a study among Swedish adolescents [22]. Similar results are found in adult populations in the US as well [5]. In US adults, Rose et al. found that GHGE diets were also associated with age and race/ethnicity, where those under 30 and non-Hispanic African Americans were more likely to consume lower GHGE diets [5]. Our study follows a similar methodological approach to Rose et al. [6] but focuses on a sample of US children rather than adults.

School meals represent a major component of the U.S. food system, with 30 million children participating daily through federally supported programs such as the National School Lunch Program and School Breakfast Program [23]. These programs provide meals to students across all income levels and play a critical role in promoting food access, particularly for those from low-income households who qualify for free or reduced-price meals based on income eligibility criteria [24]. Given their scale and policy relevance, school meals may also contribute meaningfully to dietary greenhouse gas emissions (GHGEs). One study examined the impact of school meals on GHGEs in the United Kingdom and found that the mean GHGE value for one school lunch is estimated to be 0.72 kg of CO_2_-equivalents, which equates to 579.1 million kg of CO_2_ emissions a year [25], whereas US school lunches found the mean GHGE of each meal to be 1.5 kg CO_2_-equivalent [26], demonstrating that UK school meals contribute less GHGEs than US school meals.

Because of the gaps related to understanding dietary GHGE in children in the US, the purpose of this study was to explore relationships between GHGE and dietary outcomes among US schoolchildren. The secondary purpose of this study was to examine demographic factors associated with high-GHGE diets and assess the relationship between school meals and GHGE in the US.

## 2. Materials and Methods

### 2.1. Study Sample and Dietary Intake Data

The US Department of Agriculture (USDA) School Nutrition and Meal Cost Study (SNMCS) was a cross-sectional study that took place during the 2014–2015 school year, which comprehensively looked at school meal programs in the United States. This study was selected because it has the most recent comprehensive dietary data examining the dietary intake of schoolchildren, inclusive of school meal consumption. The SNMCS is a nationally representative sample of public-school food authorities, schools, and students in the 48 contiguous states and the District of Columbia [27].

A comprehensive sampling frame was developed to minimize potential selection bias. This study sampled 548 district-level school meal providers, resulting in participation from 1282 schools. A total of 2748 24 h dietary recalls were collected from 2165 unique students, with approximately 27% completing a second recall. For this analysis, only the first-day recall from each student was used (N = 2165). Parent-assisted recalls were included for younger children. A detailed description of the SNMCS sampling methodology is available elsewhere [27].

Dietary recalls were conducted Tuesday through Saturday, with each recall assessing intake from the prior day. This approach ensured that recalled intake corresponded to school days (Monday through Friday), allowing for capture of school meal participation and consumption. Consent was obtained from parents; passive consent was used whenever possible, but active consent was required by 31 schools [27]. Data were collected using the USDA’s Automated Multiple-Pass Method [28], a computer-assisted personal interview, which was modified to include specific school locations like reimbursable cafeteria lines, vending machines, and school stores [27].

Because this study involved the analysis of deidentified, secondary data, Brigham Young University’s Institutional Review Board deemed it exempt from review.

### 2.2. Greenhouse Gas Emissions Data

Greenhouse gas emissions (GHGEs) were selected as the primary measure of environmental health in this study because of their widespread use in quantifying the environmental impact of food production [29]. This measure is quantified as kilograms of CO_2_-equivalents per kilogram of food [30], representing emissions from multiple stages of food production, including production, processing, and transportation [31]. GHGE was chosen because it is the most widely available and used metric for analyzing dietary environmental impacts [29].

Food-related GHGEs were calculated for each person by linking food items and amounts of food consumed from the SNMCS 24 h dietary recalls to dataFRIENDS (database of Food Recall Impacts on the Environment for Nutrition and Dietary Studies, version 2). DataFRIENDS compiles GHGE estimates for foods using 8-digit United States Department of Agriculture (USDA) food codes, capturing emissions related to production and primary processing. This dataset is specifically designed to integrate environmental data with US dietary records, providing GHGE values in kg CO_2_-equivalents (CO_2_-eq) per kilogram of food. Details on the creation of this database are described elsewhere [5,31]. This dataset contains the GHGE (kg CO_2_-equivalents) per 100 g of 8-digit USDA food codes used in national dietary studies [32,33]. These data were merged with the foods consumed in the student dietary recalls and summed by student to get the total dietary GHGE per 1000 kcal.

There were some SNMCS recall items that were not found in dataFRIENDS; thus, proxy items were selected to obtain GHGEs for all items consumed. Two percent of the total number of food items consumed by all students required proxy matches; in all instances, they were specific items in school meals. When a proxy item was selected, the item most closely related to the missing item was chosen. For example, “SCHOOL breaded chicken patty sandwich w/3.49 oz wgr chicken patty” (food code 100248) was an item from the SNMCS that did not have a match in dataFRIENDS, as this database does not include specific food codes for school meal items. We used “Chicken fillet (breaded, fried) sandwich” (food code 27540140) as a proxy food item. The proxy food items are in dataFRIENDS. Another example is “SCHOOL hamburger on bun, w/wgr roll w/2.4 oz beef patty” (food code 102064). This food item from the SNMCS is not found in dataFRIENDS. Therefore, we used “Hamburger, plain, on bun” (food code 27510500) as a proxy. “Hamburger, plain, on bun” (food code 27510500) is in dataFRIENDS.

### 2.3. Dietary Measures

Dietary intake was analyzed using a selection of nutrients, food groups, and the Healthy Eating Index 2010 (HEI) [34] and its components as measures of overall diet quality. The nutrients of interest were total saturated fatty acids, dietary fiber, Vitamin A, Vitamin C, Vitamin E, total choline, iron, calcium, magnesium, potassium, and sodium. At the time of data collection (2014), these were nutrients of concern according to the Dietary Guidelines for Americans 2010–2015 [35]. The 2010 version of the HEI was selected to align with the Dietary Guidelines for Americans (DGAs) in effect during the study period and to ensure consistency with the timing of the dietary data. According to the Dietary Guidelines for Americans, food group intakes were calculated using the Food Patterns Equivalents Database (FPED) [36]. The groups analyzed from the Food Patterns Equivalents Database included fruits, vegetables, refined grains, whole grains, protein foods, dairy, oils, solid fats, and added sugars. For all protein foods, these were further separated out by animal protein foods and plant protein foods. Meat, poultry, and seafood are considered animal protein foods. Legumes, soy products, nuts, and seeds are considered plant protein foods. All nutrient and food groups were reported as densities, per 1000 kcal, versus total amounts consumed. Food group intakes were expressed as standardized cup-equivalents, ounce-equivalents, and teaspoon-equivalents per 1000 kcal, consistent with the FPED and HEI methodology. For approximate conversions of these guidance-based food groups to international units, see Supplementary Table S1.

The HEI, developed as a collaborative effort between the National Cancer Institute (US Department of Health and Human Services) and the Center for Nutrition Policy and Promotion (US Department of Agriculture), is a summary measure of overall diet quality based on how well dietary intake aligns with the DGAs and helps distinguish high- and low-quality diets [34]. Higher scores on the HEI indicate a healthier diet due to greater consumption of food groups such as fruits, vegetables, whole grains, and plant proteins, while lower scores reflect poorer diet quality with higher consumption of sodium, refined grains, and empty calories.

HEI scores range from 0 to 100 and include 12 different components. The score gives more points for higher consumption of total fruits, whole fruits, total vegetables, greens and beans, whole grains, dairy, total protein foods, seafood and plant proteins, and the ratio of mono- and poly-unsaturated fatty acids to saturated fatty acids. It also gives more points for lower consumption of refined grains, sodium, and empty calories. The closer the score is to 100, the more the diet aligns with the DGA (in other words, the higher the diet quality). The simple HEI scoring algorithm was applied to calculate individual-level HEI scores [37,38], as this method is appropriate for analyzing cross-sectional dietary recall data and comparing diet quality across groups. We analyzed total HEI scores and all component scores.

### 2.4. Sociodemographic and School Meal Measures

To examine factors associated with dietary GHGE, participant characteristics, including self-reported gender, race or ethnicity, usual participation in school meals, and grade level, were collected from students, while household income was reported by parents or guardians. Gender was recorded as either male or female; the binary response option for gender presents a limitation, as it does not account for non-binary or gender-diverse individuals, but this is how the SNMCS data were collected. Race or ethnicity was categorized as Hispanic, White only (non-Hispanic), Black only (non-Hispanic), and Other, which includes multiracial individuals. The dataset categorized students into four mutually exclusive groups: ‘Hispanic,’ ‘White only’ (non-Hispanic), ‘Black only’ (non-Hispanic), and ‘Other’ (including multiracial). While the questionnaire allowed respondents to select both race and ethnicity, the final dataset coding grouped all students who identified as Hispanic into a single ‘Hispanic’ category, regardless of their reported race. The dataset calculated the ratio of reported income to US poverty guidelines and categorized students as either less than 185% or greater than or equal to 185% of the US federal poverty level. For a student to qualify for free or reduced-price school meals, their household income must be less than 185% of the poverty threshold. In this study, “school meals” refers to meals obtained through the school meal program, regardless of whether they were free, reduced-price, or full-price. School meal participation was measured as target day participation and usual participation. Target day participation indicated that students consumed a school meal on the day of the 24 h dietary recall. Students also reported how many days per week they typically consumed school meals, with separate questions for lunch and breakfast. Those reporting ≥ 3 days/week were classified as usual participants [27]. Grade level was divided into elementary (ages 5–10), middle (ages 11–13), and high school (ages 14–18). These categories generally correspond to primary (elementary) and secondary (middle and high school) education in international contexts.

### 2.5. Statistical Analysis

Respondents’ total dietary GHGE per 1000 kcal were ordered from low to high and categorized into quintiles. Nutrient intakes, food group intakes, diet quality (HEI), participation in school meals, and demographic characteristics were compared between students in the low-GHGE (Q1) and high-GHGE (Q5) groups using *t*-tests (nutrient intakes, food group intakes, HEI-2010, and GHGE) and chi-square tests (demographic characteristics and school meal participation). Detailed analyses of the complete dataset, including all quintiles, are available in the supplemental tables. Statistical significance was determined at an alpha level of 0.05 (*p* < 0.05).

In addition to total dietary GHGE, greenhouse gas emissions attributable to school-obtained foods were calculated. Foods were classified as school-obtained if they were reported as obtained from the reimbursable school cafeteria line. GHGEs from these items were summed for each student and expressed both as absolute GHGE and as a percentage of the total daily GHGE. Differences in the proportion of GHGEs attributable to school-obtained foods across quintiles were examined descriptively and compared between the lowest (Q1) and highest (Q5) quintiles using *t*-tests.

To assess whether observed differences persisted after accounting for potential confounding, multivariable analyses were conducted for total greenhouse gas emissions (logistic regression) and HEI total score and component scores (linear regression). The models were adjusted for sex, race–ethnicity, income-to-poverty ratio, grade level, and whether the student ate school meals on the day of the recall. All analyses were conducted in SAS version 9.4 (Cary, NC, USA).

## 3. Results

A total of 2165 primary and secondary schoolchildren completed dietary recalls. Participation was relatively evenly distributed across school levels, with 39.63% of participants in the high school category. Almost half of the sample (48.65%) identified as non-Hispanic White, followed by 28.13% identifying as Hispanic, 13.63% as non-Hispanic Black, and 9.60% as multiracial or Other. The gender distribution was balanced, with 51.96% being male and 48.04% being female (Table 1).

The average GHGE was 3.64 kg CO_2_-eq per person per day for the total sample. When separating the total sample into quintiles, the low-GHGE group (*n* = 433) consumed an average of 1.54 kg CO_2_-eq per person per day compared to the high-GHGE group (*n* = 433), who consumed an average of 7.37 kg CO_2_-eq per person per day (*p* < 0.0001). Supplementary Table S2 presents participant characteristics from the study sample for the entire dataset.

Statistically significant differences were observed between the high- and low-GHGE groups for several characteristics in bivariate analyses. Gender showed a marked difference, with males more likely to be in the high-GHGE group (59.16% vs. 41.54%, *p* < 0.0001). Usual participation in school lunch was also higher in the high-GHGE group (65.89% vs. 58.00%, *p* = 0.02), as was usual participation in school breakfast (30.39% vs. 21.70%, *p* = 0.005). There were no significant differences in race or ethnicity, household income, or grade level between the low- and high-GHGE groups (Table 1).

In multivariable logistic regression analyses, male students had higher odds of being in the high-GHGE group compared to female students (OR: 1.58; 95% CI: 1.18–2.11). Students who reported consuming a school meal on the day of recall also had greater odds of high GHGEs (OR: 1.40; 95% CI: 1.04–1.88). No statistically significant differences in the odds of high GHGEs were observed by race or ethnicity, poverty level, or grade level (Table 2).

Greenhouse gas emissions attributable to school-obtained foods accounted for a similar proportion of total daily GHGE in both low- and high-GHGE groups. On average, school-obtained foods contributed 10.45% (SD 18.25) of total daily GHGE in the low-GHGE group and 9.83% (SD 18.96) in the high-GHGE group; this difference was not statistically significant (*p* = 0.62). The mean absolute GHGE from school-obtained foods was 0.15 kg CO_2_-eq/day (SD 0.28) in the low-GHGE group and 0.70 kg CO_2_-eq/day (SD 1.65) in the high-GHGE group (Table 1).

Odds ratios represent the adjusted odds of being in the high-GHGE group (quintile 4) versus the low-GHGE group (quintile 1), controlling for race/ethnicity, gender, poverty level, grade level, and school meal consumption on the day of recall.

Compared to the high-GHGE group, the low-GHGE group had higher intakes of fiber and Vitamin E and lower intakes of sodium and saturated fat, whereas the high-GHGE group had higher intakes of several micronutrients, including Vitamin A, choline, calcium, magnesium, and potassium (Table 3). Supplementary Table S3 presents means and standard deviations for nutrient intakes per 1000 kcals for the entire sample.

When comparing high- and low-GHGE groups with their food consumption, there were also statistically significant differences. The low-GHGE group consumed significantly more total grains (*p* < 0.0001), including both whole grains (*p* = 0.0053) and refined grains (*p* < 0.0001), compared to the high-GHGE group (Table 4). They also consumed significantly fewer ounce-equivalents of total protein foods (*p* < 0.0001), with notable differences in the types of protein consumed. Specifically, the low-GHGE group consumed less animal protein (*p* < 0.0001), including less meat (*p* < 0.0001) and seafood (*p* = 0.0048), but more poultry (*p* < 0.0001) and significantly more plant protein (*p* < 0.001). In addition, the low-GHGE group consumed less total dairy (*p* < 0.0001) and solid fats (*p* < 0.0001) but more oils (*p* < 0.0001) and added sugars (*p* < 0.0001). While the total fruit intake (cups per 1000 kcals) did not differ significantly between groups (*p* = 0.13), the high-GHGE group consumed more vegetables (*p* < 0.0001) (Table 5). Supplementary Table S4 presents means and standard deviations for food group intakes across GHGE quintiles for the entire sample.

When comparing HEI scores, there was no significant difference in the total HEI-2010 score between the low- and high-GHGE groups (*p* = 0.65). However, several component scores differed significantly between the groups. The low-GHGE group scored significantly higher on whole fruit (*p* = 0.03), fatty acids (*p* < 0.0001), and sodium components (*p* < 0.0001), while the high-GHGE group scored significantly higher on total vegetables (*p* < 0.0001), dairy (*p* < 0.0001), total protein foods (*p* < 0.0001), seafood and plant proteins (*p* = 0.02), refined grains (*p* < 0.0001), and empty calories components (*p* = 0.006). No significant differences were observed in total fruit, greens and beans, or whole grains components (Table 5). Supplementary Table S5 presents means and standard deviations for HEI-2010 component and total scores across GHGE quintiles for the entire sample.

In multivariable analyses, the total HEI-2010 score did not differ by the GHGE group. Consistent with bivariate findings, most component-level results were unchanged. In the adjusted model, whole-grain component scores were significantly higher in the low-GHGE group, while no differences were observed for seafood and plant protein component scores. Additional adjusted results are presented in Supplementary Table S6.

## 4. Discussion

The present study identified significant differences in specific components of dietary quality between the lowest and highest dietary GHGE groups (referred to here as the low-GHGE and high-GHGE groups), although no differences were observed in the total diet quality score. Differences between groups were observed across several HEI-2010 components, reflecting variations in dietary patterns and food group composition, as well as in selected nutrient intakes, which may have implications for the overall health impact of these diets.

A significant relationship between demographic factors and GHGE was identified. In the present study, females were more likely to be in the low-GHGE group compared to males. Typically, females consume fewer calories than males; however, we standardized the GHGE per 1000 calories, and females were still more likely to be in the low-GHGE group regardless of total energy intake. This result is observed in several other studies examining dietary GHGE and gender as well, and it indicates that the diet patterns of females differ not just in amounts of food but also in the types of foods consumed [5]. Lindroos et al. found that males were more likely to have higher dietary GHGEs compared to females in a study among Swedish adolescents [22].

The average GHGE for the sample was 3.64 kg CO_2_-equivalent, which is slightly higher than the average GHGE reported in the Netherlands (3.2 kg CO_2_-equivalent) [21] and slightly lower than in Sweden (3.8–4.2 kg CO_2_-equivalent) [22]. Differences among children and adolescent dietary GHGE may be attributed to variations in food preferences, portion sizes, the availability of plant-based options, and differences in the GHGE databases used [21,22,39,40].

The present study also found a relationship between dietary GHGE and participation in the National School Lunch Program [41] and School Breakfast Program [42]. Both participation in school meals on the day of the dietary recall and usual participation (participating > 3 times/week) were associated with being in the high-GHGE group. School meals typically provide a substantial share of daily caloric intake for children, often accounting for up to one-third of their total daily energy intake [43]. However, because our analyses used GHGE per 1000 kcal, this significant contribution suggests that the higher GHGE observed among participants may be partly explained by the types of foods served, particularly those required by USDA guidelines, such as meats/meat alternatives and dairy. Meal pattern requirements are set by the USDA and specify minimum weekly amounts of these components; for example, elementary school students are typically offered approximately 8–10 ounce-equivalents of meat or meat alternatives per week at lunch, along with 5 cups of milk. Meat alternatives include foods such as beans, legumes, tofu, eggs, and nut or seed products [44,45]. Stern et al. found the mean impact of lunch in the United States was 1.5 kg CO_2_-equivalent [26], which is higher than the estimates of the mean value for school lunch in the United Kingdom (estimated to be 0.72 kg of CO_2_) [25]. Stern et al. suggest reducing environmental impacts through implementing a serving size or frequency limit for beef in the National School Lunch Program [26]. However, it is also important to note that other factors in the diets of participants, outside of school meals, could contribute to their higher GHGE, and further analysis would be needed to examine these associations. Moreover, given the cross-sectional design of this study, these findings should be interpreted as associations rather than causal relationships.

When examining nutrient intakes per 1000 kcals, the low-GHGE group had significantly higher intakes of dietary fiber and added sugars and lower intakes of saturated fat and sodium. These specific outcomes have significance for long-term health. For example, high sugar consumption among children is associated with dental decay, nutritional inadequacy, and the development of high blood pressure and lipid abnormalities [46,47,48]. Consumption of saturated fat is strongly correlated with low-density lipoproteins (LDLs) [49], and elevated LDLs in childhood are associated with an increase in cardiovascular disease risk factors in adulthood [50]. A review article by Payne et al. demonstrates that research on GHGE and dietary quality in adults shows mixed findings. Their report highlights inconsistencies, particularly regarding the association of lower GHGE diets and reduced salt and sugar intake, although many studies suggest a relationship between lower GHGE and reduced saturated fat intake [51]. Fewer studies have explored the relationship between GHGE and children’s nutrient intakes.

The low-GHGE group received better scores on the fatty acid ratio, sodium, and other components of the HEI. However, the high-GHGE group scored better on other components, such as dairy, total protein foods, and refined grains. Despite these differences in individual components, there was no significant difference in the total HEI-2010 score between groups. This finding is notable and suggests that composite measures of diet quality may obscure meaningful differences in underlying dietary patterns, and this highlights the complexity of the relationship between dietary quality and environmental sustainability. The results challenge simplified interpretations that lower-GHGE diets are inherently “healthier” and instead suggest that such diets may represent different nutritional profiles rather than uniformly better ones. Similar mixed findings have been observed in prior studies, particularly in adult populations, where lower-GHGE diets are often associated with lower saturated fat intake but a different pattern for other nutrients or food components [6,51]. One study conducted in Swedish adolescents found that higher diet quality was indeed associated with lower GHGEs [22], suggesting that these relationships may vary across populations and measurement approaches. Taken together, these findings underscore the importance of evaluating both overall diet quality and individual dietary components when assessing the health implications and environmental impacts of diets.

The present study identifies the diet-related GHGE levels for children and adolescents. Although the analysis was limited to specific quintiles of the national sample, the national scope of the dataset remains a strength of this study; this is the first study to link environmental impact data to children’s diet in the United States. Given the pressing concern for climate change across the globe, further considerations can and should be made relative to dietary intake among children and adolescents. This may include further research into interventions on ways to reduce dietary GHGE among children.

One mechanism for reducing GHGE in children and adolescents would be to focus on the school food environment, given that children consume a substantial proportion of their daily energy intake at school [43]. This study highlights how dietary intake among children and adolescents varies based on participation in school meal programs. Some school districts have made efforts to reduce GHGEs. For example, the New York City Department of Education has made plant-based dishes the primary menu items on Fridays [52]. USDA could support pilot programs that give students the option to have plant-based entrees in their schools, like what was developed in California [53]. These findings have important implications for both research and policy. The presence of nutrient-specific trade-offs alongside similar overall diet quality scores may help explain inconsistencies in the existing literature on diet-related greenhouse gas emissions (GHGEs), particularly when different studies rely on composite indices versus nutrient- or food-based measures. Our results suggest that conclusions regarding the alignment between dietary quality and GHGE may depend on how dietary quality is operationalized.

From a policy perspective, these findings indicate that efforts to promote environmentally sustainable diets in children and adolescents should move beyond broad recommendations to “reduce emissions” and instead consider the nutritional consequences of specific dietary shifts. For example, reducing high-emission foods such as certain animal-source products may lower GHGEs but could also affect intake of key nutrients such as calcium or protein if not carefully balanced. As such, dietary guidelines and school meal policies should aim to identify strategies that minimize environmental impact while preserving nutrient adequacy, particularly during critical periods of growth and development.

Despite the significant findings reported in our study, several limitations should be considered when interpreting the results. First, dietary intake was based on a single 24 h recall, which may not reflect usual intake. Thus, individuals may not have been correctly classified into high- or low-GHGE groups, which could attenuate observed associations with demographic variables. Second, the use of the simple scoring algorithm to calculate HEI-2010 may reduce precision when estimating group-level dietary quality and may not fully capture variability in intake patterns [35]. This limitation may have contributed to the lack of observed differences in total HEI scores between groups, despite differences in individual dietary components. Third, the data were collected in 2014–2015, representing the most recent nationally representative dataset that can be linked with dataFRIENDS and aligning with the time frame of foods included in that resource. Although dietary patterns and food systems may have shifted since that time—such as increased availability of plant-based alternatives or evolving school meal standards—these data remain informative for understanding population-level dietary patterns and their associated GHGEs. Additionally, this study reflects the U.S. food system, including national dietary patterns, food fortification practices, and federally regulated school meal programs, which may limit generalizability to other countries with different food systems and policies. Fourth, this study focused exclusively on greenhouse gas emissions (GHGEs) as a measure of environmental impact. While GHGE is a major contributor to climate change, it does not capture other important dimensions such as water use, land use, or biodiversity loss. As a result, the environmental implications of dietary patterns observed in this study may be incomplete. Finally, the use of proxy values for some food items may introduce measurement error in the estimated GHGE. Although proxy values were used for a very small proportion of food items, this approach could lead to over- or underestimation of emissions for certain foods, depending on how closely proxy items reflect actual consumption. Despite these limitations, this study provides important insights into the relationship between dietary patterns and greenhouse gas emissions among U.S. schoolchildren.

## 5. Conclusions

Food patterns of students with lower dietary GHGE were more nutritious on some key dimensions and less nutritious on others, with no difference observed in overall diet quality between groups. These findings suggest that reducing dietary GHGEs may be possible without substantially altering overall diet quality, but this is accompanied by trade-offs across specific nutrients and food groups. Participation in school meal programs was associated with higher GHGEs; however, further research is needed to better understand the underlying drivers of this relationship.

Efforts to reduce dietary GHGE in children and adolescents should consider both environmental and nutritional outcomes, with particular attention to tradeoffs between the two, to maintain adequate intake of key nutrients essential for growth and development. These findings can inform policies and programs aimed at improving dietary quality while also addressing environmental sustainability.

## Figures and Tables

**Table 1:** Participant characteristics of the study sample and the US School Nutrition and Meal Cost Study 2014–2015.

Variable	Overall Sample N = 2165 N (%)	Low-Greenhouse-Gas-Emission Diet *n* = 433 *n* (%)	High-Greenhouse-Gas-Emission Diet *n* = 433 *n* (%)	*p*-Value ^d^
Gender				<0.0001
Male	1113 (52.0)	177 (41.8)	255 (59.2)	
Female	1029 (48.0)	246 (58.2)	176 (40.8)	
Missing	23	10	2	
Race or ethnicity	551 (28.1)	106 (28.8)	108 (26.8)	0.44
Hispanic	953 (48.7)	183 (49.7)	189 (46.9)	
Non-Hispanic White	267 (13.6)	48 (13.0)	61 (15.1)	
Non-Hispanic Black	188 (9.6)	31 (8.4)	45 (11.2)	
Other (including multiracial)	206	65	30	
Poverty threshold ^a^				0.55
≤185% of poverty threshold	1004 (47.3)	191 (45.5)	203 (47.5)	
>185% of poverty threshold	1119 (52.7)	229 (54.5)	224 (52.5)	
Missing	42	13	6	
Grade				0.31
Elementary (primary)	668 (30.9)	108 (24.9)	128 (29.6)	
Middle (secondary)	639 (29.5)	140 (32.4)	133 (30.7)	
High (secondary)	858 (39.6)	185 (42.7)	172 (39.7)	
Missing	0	0	0	
Usual school lunch participation ^b^				0.02
No	803 (37.2)	181 (42.0)	147 (34.1)	
Yes	1355 (62.8)	250 (58.0)	284 (65.9)	
Missing	7	2	2	
Usual school breakfast participation ^b^				0.005
No	1456 (72.4)	314 (78.3)	284 (69.6)	
Yes	554 (27.6)	87 (21.7)	124 (30.4)	
Missing	155	32	25	
Ate school meals the day of recall				<0.0001
No	1038 (48.0)	251 (57.97)	152 (35.1)	
Yes	1127 (52.0)	182 (42.03)	281 (64.9)	
Missing	0	0	0	
	**Mean (SD)**	**Mean (SD)**	**Mean (SD)**	
Total greenhouse gas (kg CO_2_-eq) ^c^	3.64 (2.93)	1.54 (0.78)	7.37 (3.97)	<0.0001
Greenhouse gas emissions from foods obtained at school (kg CO_2_-eq) ^c^	0.39 (0.90)	0.15 (0.28)	0.70 (1.65)	<0.0001

^a^ The US federal poverty threshold is based on the household size and income. If a household’s total income is less than the threshold for its size, the household is considered to be in poverty. ^b^ Students who participate in the school lunch or breakfast program at least three times per week are considered usual participants. ^c^ CO_2_-eq is carbon dioxide equivalent. ^d^ *p*-values were calculated using chi-square tests for categorical variables and independent samples *t*-tests for continuous variables.

**Table 2:** Multivariable logistic regression of high vs. low dietary greenhouse gas emissions by demographic characteristics.

Variable	Odds Ratio (95% CI)	*p*-Value
Gender		0.002
Female	Ref.	
Male	1.58 (1.18, 2.11)	
Race or ethnicity		0.23
White	Ref.	
Hispanic	1.22 (0.86, 1.85)	
Non-Hispanic Black	1.19 (0.75, 1.90)	
Other	1.64 (1.00, 2.68)	
Poverty level		0.56
>185% of poverty threshold	Ref.	
≤185% of poverty threshold	1.09 (0.80, 1.49)	
Grade		0.05
High school (secondary)	Ref.	
Middle school (secondary)	0.75 (0.53, 1.07)	
Elementary school (primary)	1.21 (0.85, 1.70)	
Ate school meals the day of recall		0.03
No	Ref.	
Yes	1.40 (1.04, 1.88)	

**Table 3:** Nutrient intake per 1000 kilocalories in low- and high-greenhouse-gas-emission (GHGE) diets: findings from the 2014–2015 US School Nutrition and Meal Cost Study (SNMCS).

Nutrient	Low-Greenhouse-Gas-Emission Diet *n* = 433 Mean (SD)	High-Greenhouse-Gas-Emission Diet *n* = 433 Mean (SD)	*p*-Value ^b^
GHGE, kg CO_2_ eq/1000 kcal	0.8 (0.2)	3.7 (1.3)	<0.0001
Dietary fiber, g/1000 kcal	9.6 (4.0)	8.3 (3.1)	<0.0001
Vitamin A, mcg RAE ^a^/1000 kcal	261 (218)	337 (356)	0.0002
Vitamin C, mg/1000 kcal	41.2 (38.8)	41.0 (36.6)	0.94
Vitamin D (D_2_ + D_3_), mcg/1000 kcal	3.7 (9.4)	3.9 (4.7)	0.69
Vitamin E as α-tocopherol, mg/1000 kcal	4.4 (3.0)	3.4 (1.6)	<0.0001
Total choline, mg/1000 kcal	102.1 (37.6)	160.9 (58.4)	<0.0001
Iron, mg/1000 kcal	7.8 (3.8)	8.1 (3.0)	0.17
Calcium, mg/1000 kcal	443 (191)	598 (243)	<0.0001
Magnesium, mg/1000 kcal	134.6 (42.2)	141.7 (38.9)	0.0281
Potassium, mg/1000 kcal	1092 (315)	1350 (346)	<0.0001
Sodium, mg/1000 kcal	1468 (387)	1771 (559)	<0.0001
Total saturated fatty acids, g/1000 kcal	10.3 (3.6)	13.1 (3.8)	<0.0001

^a^ Retinol activity equivalents. ^b^ Differences in mean values between low- and high-GHGE groups were assessed using independent samples *t*-tests.

**Table 4:** Food group intakes in low- and high-greenhouse-gas-emission (GHGE) diets: findings from the 2014–2015 US School Nutrition and Meal Cost Study (SNMCS).

Food Group	Unit	Low-Greenhouse-Gas-Emission Diet *n* = 433 Mean (SD)	High-Greenhouse-Gas-Emission Diet *n* = 433 Mean (SD)	*p*-Value ^e^
Total fruit and vegetables ^a^	cup eq/1000 kcal	1.2 (0.94)	1.3 (0.9)	0.13
Fruit	cup eq/1000 kcal	1.3 (1.2)	1.3 (1.3)	0.95
Vegetables ^a^	cup eq/1000 kcal	0.4 (0.4)	0.6 (0.5)	<0.0001
Total grains	oz eq/1000 kcal	4.1 (1.5)	3.4 (1.2)	<0.0001
Whole grains	oz eq/1000 kcal	0.9 (0.9)	0.7 (0.7)	0.0053
Refined grains	oz eq/1000 kcal	3.3 (1.5)	2.6 (1.2)	<0.0001
Protein foods: total ^b^	oz eq/1000 kcal	1.8 (1.4)	3.3 (1.7)	<0.0001
Animal protein	oz eq/1000 kcal	1.0 (1.2)	2.4 (1.5)	<0.0001
Meat ^c^	oz eq/1000 kcal	0.1 (0.3)	1.8 (1.4)	<0.0001
Poultry	oz eq/1000 kcal	0.9 (1.2)	0.4 (0.8)	<0.0001
Seafood	oz eq/1000 kcal	0.1 (0.4)	0.2 (0.7)	0.0048
Plant protein ^d^	oz eq/1000 kcal	0.7 (1.1)	0.3 (0.7)	<0.0001
Total dairy	cup eq/1000 kcal	0.7 (0.5)	1.2 (0.7)	<0.0001
Oils	g/1000 kcal	14.2 (8.2)	9.5 (5.8)	<0.0001
Solid fats	g/1000 kcal	13.5 (7.5)	17.1 (7.6)	<0.0001
Added sugars	tsp eq/1000 kcal	18.8 (14.0)	14.6 (11.0)	<0.0001

^a^ Vegetable totals do not include legumes. ^b^ The total protein foods group is a sum of animal and plant protein foods. ^c^ The meat group includes beef, veal, other ruminant animals, pork, and game. ^d^ The plant protein foods group includes all legumes, soybeans, nuts, and seeds. ^e^ Differences in mean values between low- and high-GHGE groups were assessed using independent samples *t*-tests.

**Table 5:** Healthy Eating Index (HEI-2010): ^a^ component and total Scores in low- and high- greenhouse-gas-emission (GHGE) diets: findings from the 2014–2015 US School Nutrition and Meal Cost Study (SNMCS).

HEI Component	Maximum Score	Low-Greenhouse-Gas-Emission Diet *n* = 433 Mean (SD)	High-Greenhouse-Gas-Emission Diet *n* = 433 Mean (SD)	*p*-Value ^d^
Total fruit	5	3.0 (2.0)	2.9 (2.0)	0.25
Whole fruit	5	3.0 (2.2)	2.7 (2.2)	0.03
Total vegetables	5	2.1 (1.6)	2.5 (1.6)	<0.0001
Greens and beans	5	0.6 (1.6)	0.6 (1.5)	0.81
Whole grains	10	4.8 (3.9)	4.3 (3.7)	0.06
Dairy	10	5.3 (3.4)	7.5 (3.0)	<0.0001
Total protein foods	5	3.3 (1.7)	4.6 (0.7)	<0.0001
Seafood and plant proteins	5	0.8 (1.8)	1.1 (1.9)	0.02
Fatty Acids	10	6.6 (3.6)	3.7 (3.2)	<0.0001
Refined grains ^b^	10	4.7 (3.7)	6.3 (3.5)	<0.0001
Sodium ^b^	10	5.9 (3.3)	3.6 (3.2)	<0.0001
Empty calories ^b,c^	20	17.3 (4.2)	18.0 (3.3)	0.006
Total HEI score	100	57.4 (13.0)	57.7 (11.0)	0.65

^a^ HEI-2010 component scores are density-based (per 1000 kcal), reflecting dietary composition rather than absolute intake. ^b^ Higher component scores are considered beneficial. Thus, for refined grains, sodium, and empty calories, higher scores indicate diets that contain less of these items. ^c^ Calories from solid fats and added sugars. ^d^ Differences in mean values between low- and high-GHGE groups were assessed using independent samples *t*-tests.

## Data Availability

The School Nutrition and Meal Cost Study is publicly available data and can be found here: https://www.fns.usda.gov/research/school-meals/nutrition-meal-cost-study (accessed on 10 March 2026). DataFRIENDS is also available and can be requested through this website: https://sph.tulane.edu/sbps/nutrition/diet-environmental-impacts (accessed on 10 March 2026).

## Supplementary Materials

The following supporting information can be downloaded at https://www.mdpi.com/article/10.3390/nu18101529/s1. Table S1: Approximate Household Equivalents for FPED Units [54]; Table S2: Participant Characteristics of the Study Sample Across All Quintiles, US School Nutrition and Meal Cost Study 2014–2015; Table S3: Nutrient Intakes per 1000 Kilocalories Across Greenhouse Gas Emission (GHGE) Quintiles: Findings from the 2014–2015 US School Nutrition and Meal Cost Study (SNMCS); Table S4: Food Group Intakes Across Greenhouse Gas Emission (GHGE) Quintiles: Findings from the 2014–2015 US School Nutrition and Meal Cost Study (SNMCS); Table S5: Healthy Eating Index (HEI-2010) Component and Total Scores Across Greenhouse Gas Emission (GHGE) Quintiles: Findings from the 2014–2015 US School Nutrition and Meal Cost Study (SNMCS); Table S6: Adjusted Healthy Eating Index (HEI-2010) Component and Total Scores in Low- and High–Greenhouse Gas Emission (GHGE) Diets: Findings from the 2014–2015 US School Nutrition and Meal Cost Study (SNMCS).

## Abbreviations

The following abbreviations are used in this manuscript:

GHGEs	Greenhouse Gas Emissions
SNMCS	School Nutrition and Meal Cost Study
US	United States
USDA	United States Department of Agriculture
dataFRIENDS	Database of Food Recall Impacts on the Environment for Nutrition and Dietary Studies
HEI	Healthy Eating Index
FPED	Food Patterns Equivalents Database
DGA	Dietary Guidelines for Americans
LDL	Low-Density Lipoprotein

## References

[1] NOAA National Center for Environment Information (2023). Global Climate Report. https://www.ncei.noaa.gov/access/monitoring/monthly-report/global/202313.

[2] Rocque R.J., Beaudoin C., Ndjaboue R., Cameron L., Poirier-Bergeron L., Poulin-Rheault R.A., Fallon C., Tricco A.C., Witteman H.O. (2021). Health effects of climate change: An overview of systematic reviews. BMJ Open.

[3] Crippa M., Solazzo E., Guizzardi D., Monforti-Ferrario F., Tubiello F.N., Leip A. (2021). Food systems are responsible for a third of global anthropogenic GHG emissions. Nat. Food.

[4] Tubiello F.N., Rosenzweig C., Conchedda G., Karl K., Gütschow J., Xueyao P., Obli-Laryea G., Wanner N., Qiu S.Y., De Barros J. (2021). Greenhouse gas emissions from food systems: Building the evidence base. Environ. Res. Lett..

[5] Rose D., Heller M.C., Willits-Smith A.M., Mejer R.J. (2019). Carbon footprint of self-selected US diets: Nutritional, demographic, and behavioral correlates. Am. J. Clin. Nutr..

[6] Poore J., Nemecek T. (2018). Reducing food’s environmental impacts through producers and consumers. Science.

[7] Willett W., Rockström J., Loken B., Springmann M., Lang T., Vermeulen S., Garnett T., Tilman D., DeClerck F., Wood A. (2019). Food in the Anthropocene: The EAT–Lancet Commission on healthy diets from sustainable food systems. Lancet.

[8] Rose D., Heller M.C., Roberto C.A. (2019). Position of the Society for Nutrition Education and Behavior: The importance of including environmental sustainability in dietary guidance. J. Nutr. Educ. Behav..

[9] Pollock B.D., Willits-Smith A.M., Heller M.C., Bazzano L.A., Rose D. (2022). Do diets with higher carbon footprints increase the risk of mortality? A population-based simulation study using self-selected diets from the USA. Public Health Nutr..

[10] Samtiya M., Aluko R.E., Dhewa T., Moreno-Rojas J.M. (2021). Potential health benefits of plant food-derived bioactive components: An overview. Foods.

[11] Bechthold A., Boeing H., Schwedhelm C., Hoffmann G., Knüppel S., Iqbal K., de Henauw S., Michels N., Devleesschauwer B., Schlesinger S. (2019). Food groups and risk of coronary heart disease, stroke and heart failure: A systematic review and dose-response meta-analysis of prospective studies. Crit. Rev. Food Sci. Nutr..

[12] Qian F., Liu G., Hu F.B., Bhupathiraju S.N., Sun Q. (2019). Association between plant-based dietary patterns and risk of type 2 diabetes: A systematic review and meta-analysis. JAMA Intern. Med..

[13] Bouvard V., Loomis D., Guyton K.Z., Grosse Y., El Ghissassi F., Benbrahim-Tallaa L., Guha N., Mattock H., Straif K. (2015). International Agency for Research on Cancer Monograph Working Group. Carcinogenicity of consumption of red and processed meat. Lancet Oncol..

[14] Wang X., Lin X., Ouyang Y.Y., Liu J., Zhao G., Pan A., Hu F.B. (2016). Red and processed meat consumption and mortality: Dose-response meta-analysis of prospective cohort studies. Public Health Nutr..

[15] Wolk A. (2017). Potential health hazards of eating red meat. J. Int. Med..

[16] Appannah G., Murray K., Trapp G., Dymock M., Oddy W.H., Ambrosini G.L. (2021). Dietary pattern trajectories across adolescence and early adulthood and their associations with childhood and parental factors. Am. J. Clin. Nutr..

[17] Movassagh E.Z., Baxter-Jones A.D.G., Kontulainen S., Whiting S.J., Vantanparast H. (2017). Tracking dietary patterns over 20 years from childhood through adolescence into young adulthood: The Saskatchewan pediatric bone mineral accrual study. Nutrients.

[18] Tan C.C., Ruhl H., Chow C.M., Ellis L. (2016). Retrospective reports of parental feeding practices and emotional eating in adulthood: The role of food preoccupation. Appetite.

[19] Conrad Z., Thorne-Lyman A.L., Wu S., DiStaso C., Korol M., Love D.C. (2025). Are healthier diets more sustainable? A cross-sectional assessment of 8 diet quality indexes and 7 sustainability metrics. Am. J. Clin. Nutr..

[20] Conrad Z., Blackstone N.T., Roy E.D. (2020). Healthy diets can create environmental trade-offs, depending on how diet quality is measured. Nutr. J..

[21] Temme E.H.M., Toxopeus I.B., Kramer G.F., Brosens MCCDrijvers J.M., Tyszler M., Ocké M.C. (2015). Greenhouse gas emissions of diets in the Netherlands and associations with food, energy and macronutrient intakes. Public Health Nutr..

[22] Lindroos A.K., Hallstrom E., Moreaus L., Strid A., Winkvist A. (2023). Dietary greenhouse gas emissions and diet quality in a cross-sectional study of Swedish adolescents. Am. J. Clin. Nutr..

[23] U.S. Department of Agriculture, Food and Nutrition Service Child Nutrition Tables. https://www.fns.usda.gov/pd/child-nutrition-tables.

[24] U.S. Department of Agriculture, Economic Research Service National School Lunch Program. https://www.ers.usda.gov/topics/food-nutrition-assistance/child-nutrition-programs/national-school-lunch-program.

[25] Wickramsinghe K.K., Rayner M., Goldacre M., Townsend N., Scarborough P. (2016). Contribution of healthy and unhealthy primary school meals to greenhouse gas emissions in England: Linking nutritional data and greenhouse gas emission data of diets. Eur. J. Clin. Nutr..

[26] Stern A.L., Blackstone N.T., Economos C.D., Griffin T.S. (2022). Less animal protein and more whole grain in US school lunches could greatly reduce environmental impacts. Commun. Earth Environ..

[27] USDA Food and Nutrition Service School Nutrition and Meal Cost Study. https://fns-prod.azureedge.us/sites/default/files/resource-files/SNMCS-Methods-Report.pdf.

[28] Moshfegh A.J., Rhodes D.G., Baer D.J., Murayi T., Clemens J.C., Rumpler W.V., Paul D.R., Sebastian R.S., Kuczynski K.J., Ingwersen L.A. (2008). The US Department of Agriculture Automated Multiple-Pass Method reduces bias in the collection of energy intakes. Am. J. Clin. Nutr..

[29] Jones A.D., Hoey L., Blesh J., Miller L., Green A., Shapiro L.F. (2016). A systematic review of the measurement of sustainable diets. Adv. Nutr..

[30] Frank S.M., Jaacks L.M., Meyer K., Rose D., Adair L.S., Avery C.L., Taillie L.S. (2024). Dietary quality and dietary greenhouse gas emissions in the USA: A comparison of the planetary health diet index, healthy eating index-2015, and dietary approaches to stop hypertension. Int. J. Behav. Nutr. Phys. Act..

[31] Ritchie H., Rosado P., Roser M. (2022). Environmental Impacts of Food Production. https://ourworldindata.org/environmental-impacts-of-food.

[32] Heller M.C., Willits-Smith A., Meyer R., Keoleian G.A., Rose D. (2018). Greenhouse gas emissions and energy use associated with production of individual self-selected US diets. Environ. Res. Lett..

[33] US Department of Agriculture, Agricultural Research Service (2020). Food and Nutrient Database for Dietary Studies 2017–2018.

[34] US Department of Agriculture Healthy Eating Index (HEI). https://www.fns.usda.gov/cnpp/healthy-eating-index-hei.

[35] US Department of Agriculture, U.S. Department of Health and Human Services (2010). Dietary Guidelines for Americans.

[36] USDA Food Patterns Equivalents Database. https://www.ars.usda.gov/northeast-area/beltsville-md-bhnrc/beltsville-human-nutrition-research-center/food-surveys-research-group/docs/fped-overview/.

[37] Healthy Eating Index (HEI) Methods and Calculations National Cancer Institute. https://epi.grants.cancer.gov/hei/hei-methods-and-calculations.html.

[38] Kirkpatrick S.I. (2018). Applications of the Healthy Eating Index for surveillance, epidemiology, and intervention research: Considerations and caveats. J. Acad. Nutr. Diet..

[39] Eustachio Colombo P., Schäfer Elinder L., Lindroos A.K., Parlesak A. (2021). Designing nutritionally adequate and climate-friendly diets for omnivorous, pescatarian, vegetarian, and vegan adolescents in Sweden using linear optimization. Nutrients.

[40] Clune S., Crossin E., Verghese K. (2017). Systematic review of greenhouse gas emissions for different fresh food categories. J. Clean. Prod..

[41] US Department of Agriculture, Food and Nutrition Service (2021). National School Lunch Program. https://www.fns.usda.gov/nslp.

[42] US Department of Agriculture, Food and Nutrition Service (2021). School Breakfast Program. https://www.fns.usda.gov/sbp.

[43] Gordon A.R., Cohen R., Crepinsek M.K., Fox M.K., Hall J., Zeidman E. (2009). The third School Nutrition Dietary Assessment Study: Background and study design. J. Am. Diet. Assoc..

[44] USDA Food and Nutrition Service National School Lunch Program Meal Pattern Chart. https://www.fns.usda.gov/nslp/national-school-lunch-program-meal-pattern-chart.

[45] USDA Food and Nutrition Service National School Breakfast Program Meal Pattern Chart. https://www.fns.usda.gov/sbp/meal-pattern-chart.

[46] Herrick K.A., Fryar C.D., Hamner H.C., Park S., Ogden C.L. (2020). Added sugars intake among US infants and toddlers. J. Acad. Nutr. Diet..

[47] Keller A., Della Torre S.B. (2015). Sugar-sweetened beverages and obesity among children and adolescents: A review of systematic literature reviews. Child. Obes..

[48] Elfassy T., Adjoian T., Lent M. (2019). Sugary drink consumption among NYC children, youth, and adults: Disparities persist over time, 2007–2015. J. Commun. Health.

[49] Mensink R. (2016). Effects of Saturated Fatty Acids on Serum Lipids and Lipoproteins: A Systematic Review and Regression Analysis.

[50] Srinivasan S.R., Frontini M.G., Xu J., Berenson G.S. (2006). Utility of childhood non-high-density lipoprotein cholesterol levels in predicting adult dyslipidemia and other cardiovascular risks: The Bogalusa Heart Study. Pediatrics.

[51] Payne C.L., Scarborough P., Cobiac L. (2016). Do low-carbon-emission diets lead to higher nutritional quality and positive health outcomes? A systematic review of the literature. Public Health Nutr..

[52] NYC Department of Education Plant-Powered Meals. https://www.schools.nyc.gov/school-life/food/school-meals/plant-powered.

[53] California Department of Education Plant-Based Meal Options in CNPs. https://www.cde.ca.gov/ls/nu/he/vegmealoptionscnp.asp.

[54] USDA Food Patterns Equivalents Database (FPED) Methodology (2015–2016). https://www.ars.usda.gov/northeast-area/beltsville-md-bhnrc/beltsville-human-nutrition-research-center/food-surveys-research-group/docs/fped-methodology.

