# Supplementary material for: A Cross-Sectional Study of the Dietary Carbon Footprints of US Schoolchildren
Source: Nutrients. 2026 May 12;18(10):1529. doi: 10.3390/nu18101529 (PMC13209416; doi:10.3390/nu18101529)
Supplement: Supplementary file 1 [file nutrients-18-01529-s001.zip › Supplementary Table S1.docx]

**Supplementary Table S1.** Approximate Household Equivalents for FPED Units

| **Food Group** | **FPED Unit** | **Approximate Household Equivalent** | **Notes** |
| --- | --- | --- | --- |
| **Fruit** | Cup eq | 240 mL 100% fruit juice110–190 g fresh fruit45–90 g dried fruit |  |
| **Vegetables** | Cup eq | 70–150 g raw vegetables125–180 g cooked vegetables |  |
| **Grains** | Oz eq | 16 g flour28 g grain |  |
| **Dairy ^c^** | Cup eq | 240 mL milk or yogurt14–28 g hard cheese125 g soft cheese | a |
| **Meat and poultry** | Oz eq | 28 g lean meat | a |
| **Fish/seafood** | Oz eq | 28 g lean fish | b |
| **Eggs** | Oz eq | 50 g | a |
| **Nuts and seeds** | Oz eq | 14–16 g | b |
| **Legumes (beans and peas)** | Oz eq | 43 g cooked |  |
| **Added sugars** | Tsp eq | 4.2 g |  |

a Fat above allowable limit is allocated to the solid fats group.
b Fat above allowable limit is allocated to the oils group.
c Dairy includes calcium-fortified soy milk.

Reference:

USDA Food Patterns Equivalents Database (FPED) methodology (2015–2016). Available at: [https://www.ars.usda.gov/northeast-area/beltsville-md-bhnrc/beltsville-human-nutrition-research-center/food-surveys-research-group/docs/fped-methodology](https://www.ars.usda.gov/northeast-area/beltsville-md-bhnrc/beltsville-human-nutrition-research-center/food-surveys-research-group/docs/fped-methodology/)
