# Supplementary material for: A Cross-Sectional Study of the Dietary Carbon Footprints of US Schoolchildren
Source: Nutrients. 2026 May 12;18(10):1529. doi: 10.3390/nu18101529 (PMC13209416; doi:10.3390/nu18101529)
Supplement: Supplementary file 1 [file nutrients-18-01529-s001.zip › Supplementary Table S2.docx]

**Supplementary Table S2.** Participant Characteristics of the Study Sample Across All Quintiles, US School Nutrition and Meal Cost Study 2014-2015.

| **Variable** | **Quintile 1 (Low Greenhouse Gas Emission diet)**  **n=433**  **n (%)** | **Quintile 2**  **n=433**  **n (%)** | **Quintile 3**  **n=433**  **n (%)** | **Quintile 4**  **n=433**  **n (%)** | **Quintile 5 (High Greenhouse Gas Emission diet)**  **n=433**  **n (%)** |
| --- | --- | --- | --- | --- | --- |
| **Gender**  Male, %  Female, %  Missing | 177 (41.8)  246 (58.2)  10 | 237 (55.2)  192 (44.8)  4 | 218 (50.9)  210 (49.0)  5 | 226 (52.4)  205 (47.6)  2 | 255 (59.2)  176 (40.8)  2 |
| **Race or ethnicity**  Hispanic, %  Non-Hispanic White, %  Non-Hispanic Black, %  Other (including multiracial)  Missing | 106 (28.8)  183 (49.7)  48 (13.0)  31 (8.4)  65 | 110 (28.1)  198 (50.6)  48 (12.3)  35 (8.9)  42 | 116 (29.4)  175 (44.4)  62 (15.7)  41 (10.4)  39 | 111 (27.5)  208 (51.6)  48 (11.9)  36 (8.9)  30 | 108 (27.8)  189 (46.9)  61 (15.1)  45 (11.2)  30 |
| **Poverty threshold^a^**  ≤185% of poverty threshold  >185% of poverty threshold  Missing | 191 (45.5)  229 (54.5)  13 | 203 (48.3)  217 (51.7)  13 | 207 (48.5)  220 (51.5)  6 | 200 (46.6)  229 (53.4)  4 | 203 (47.5)  224 (52.5)  6 |
| **Grade**  Elementary (primary)  Middle (secondary)  High (secondary)  Missing | 108 (24.9)  140 (32.3)  185 (42.7)  0 | 128 (29.6)  123 (28.4)  182 (42.0)  0 | 143 (33.0)  124 (28.6)  166 (38.3)  0 | 161 (37.2)  119 (27.5)  153 (35.3)  0 | 128 (29.6)  133 (30.7)  172 (39.7)  0 |
| **Usual school lunch participation ^b^**  No  Yes  Missing | 181 (42.0)  250 (58.0)  2 | 155 (36.0) 276 (64.0)  2 | 157 (36.3)  275 (63.7)  1 | 163 (37.6)  270 (62.4)  0 | 147 (34.1)  284 (65.9)  2 |
| **Usual school breakfast participation ^b^**  No  Yes  Missing | 314 (78.3)  87 (21.7)  32 | 293 (72.4)  112 (27.5)  28 | 282 (70.5)  118 (29.5)  33 | 283 (71.5)  113 (28.5)  37 | 284 (69.6)  124 (30.4)  25 |
| **Ate school meals the day of recall**  No  Yes Missing | 251 (58.0)  182 (42.0)  0 | 239 (55.2)  194 (44.8)  0 | 205 (47.3)  228 (52.7)  0 | 191 (44.1)  242 (55.9)  0 | 152 (35.1)  281 (64.9)  0 |
|  | **Mean (SD)** | **Mean (SD)** | **Mean (SD)** | **Mean (SD)** | **Mean (SD)** |
| **Total greenhouse gas (kg**  **CO2-eq) ^c^** | 1.54 (0.78) | 2.38 (0.99) | 2.89 (1.20) | 3.99 (1.91) | 7.37 (3.97) |

^a^ The federal poverty threshold is based on the household size and income. If a household’s total income is less than the threshold for its size, the household is considered to be in poverty.

^b^ Students who participate in the school lunch or breakfast program at least three times per week are considered usual participants.

^c^ CO2-eq is carbon dioxide equivalent.
