# Supplementary material for: A Cross-Sectional Study of the Dietary Carbon Footprints of US Schoolchildren
Source: Nutrients. 2026 May 12;18(10):1529. doi: 10.3390/nu18101529 (PMC13209416; doi:10.3390/nu18101529)
Supplement: Supplementary file 1 [file nutrients-18-01529-s001.zip › Supplementary Table S3.docx]

**Supplementary Table S3.** Nutrient Intakes per 1,000 Kilocalories Across Greenhouse Gas Emission (GHGE) Quintiles: Findings from the 2014-2015 US School Nutrition and Meal Cost Study (SNMCS).

| **Nutrient** | **Quintile 1**  **(Low Greenhouse Gas Emission diet)**  **n=433**  **Mean (SD)** | **Quintile 2**  **n=433**  **Mean (SD)** | **Quintile 3**  **n=433**  **Mean (SD)** | **Quintile 4**  **n=433**  **Mean (SD)** | **Quintile 5**  **(High Greenhouse Gas Emission diet)**  **n=433**  **Mean (SD)** |
| --- | --- | --- | --- | --- | --- |
| GHGE, kg CO_2_ eq/1000 kcal | 0.8 (0.2) | 1.2 (0.1) | 1.5 (0.1) | 2.0 (0.2) | 3.7 (1.3) |
| Dietary fiber, g/1000 kcal | 9.6 (4.0) | 9.0 (3.7) | 9.0 (3.5) | 8.7 (3.5) | 8.3 (3.1) |
| Vitamin A, mcg RAE ^a^/1000 kcal | 261 (218) | 335 (225) | 368 (233) | 338 (226) | 337 (356) |
| Vitamin C, mg/1000 kcal | 41.2 (38.8) | 43.2 (37.3) | 43.9 (39.7) | 46.5 (44.8) | 41.0 (36.6) |
| Vitamin D (D_2_ + D_3_), mcg/1000 kcal | 3.7 (9.4) | 4.2 (6.1) | 4.9 (5.9) | 4.9 (9.0) | 3.9 (4.7) |
| Vitamin E as α-tocopherol, mg/1000 kcal | 4.4 (3.0) | 4.1 (2.5) | 3.7 (2.2) | 3.6 (2.0) | 3.4 (1.6) |
| Total choline, mg/1000 kcal | 102.1 (37.6) | 127.4 (48.4) | 143.9 (51.1) | 146.1 (60.2) | 160.9 (58.4) |
| Iron, mg/1000 kcal | 7.8 (3.8) | 8.0 (4.3) | 8.1 (3.7) | 7.2 (3.1) | 8.1 (3.0) |
| Calcium, mg/1000 kcal | 443 (191) | 558 (205) | 649 (238) | 615 (271) | 598 (243) |
| Magnesium, mg/1000 kcal | 134.6 (42.2) | 141.3 (39.3) | 147.3 (37.6) | 142.9 (40.0) | 141.7 (38.9) |
| Potassium, mg/1000 kcal | 1092 (315) | 1205 (322) | 1323 (357) | 1339 (381) | 1350 (346) |
| Sodium, mg/1000 kcal | 1468 (387) | 1527 (402) | 1630 (416) | 1672 (420) | 1771 (559) |
| Total saturated fatty acids, g/1000 kcal | 10.3 (3.6) | 11.4 (3.5) | 11.6 (3.5) | 12.3 (3.7) | 13.1 (3.8) |

^a^ Retinol Activity Equivalents
