# Supplementary material for: A Cross-Sectional Study of the Dietary Carbon Footprints of US Schoolchildren
Source: Nutrients. 2026 May 12;18(10):1529. doi: 10.3390/nu18101529 (PMC13209416; doi:10.3390/nu18101529)
Supplement: Supplementary file 1 [file nutrients-18-01529-s001.zip › Supplementary Table S4.docx]

**Supplementary Table S4.** Food Group Intakes Across Greenhouse Gas Emission (GHGE) Quintiles: Findings from the 2014-2015 US School Nutrition and Meal Cost Study (SNMCS).

| **Food group** | **Unit** | **Quintile 1**  **Low Greenhouse Gas Emission diet**  **n=433**  **Mean (SD)** | **Quintile 2**  **n=433**  **Mean (SD)** | **Quintile 3**  **n=433**  **Mean (SD)** | **Quintile 4**  **n=433**  **Mean (SD)** | **High Greenhouse Gas Emission diet**  **n=433**  **Mean (SD)** |
| --- | --- | --- | --- | --- | --- | --- |
| Total fruit and vegetables ^a^  Fruit  Vegetables ^a^ | cup eq/1000 kcal  cup eq/1000 kcal  cup eq/1000 kcal | 1.2 (0.9)  1.3 (1.2)  0.4 (0.4) | 1.2 (0.9)  1.5 (1.5)  0.5 (0.5) | 1.3 (1.0)  1.4 (1.2)  0.5 (0.5) | 1.3 (0.9)  1.4 (1.4)  0.6 (0.5) | 1.3 (0.9)  1.3 (1.3)  0.6 (0.5) |
| Total grains  Whole grains  Refined grains | oz eq/1000 kcal  oz eq/1000 kcal  oz eq/1000 kcal | 4.1 (1.5)  0.9 (0.9)  3.3 (1.5) | 3.9 (1.3)  0.9 (0.9)  3.1 (1.3) | 3.8 (1.3)  0.9 (0.8)  2.9 (1.3) | 3.4 (1.2)  0.7 (0.7)  2.6 (1.2) | 3.4 (1.2)  0.7 (0.7)  2.6 (1.2) |
| Protein foods: total ^b^  Animal protein  Meat ^c^  Poultry  Seafood  Plant protein ^d^ | oz eq/1000 kcal  oz eq/1000 kcal  oz eq/1000 kcal  oz eq/1000 kcal  oz eq/1000 kcal  oz eq/1000 kcal | 1.8 (1.4)  1.0 (1.2)  0.1 (0.3)  0.9 (1.2)  0.1 (0.4)  0.7 (1.1) | 2.1 (1.4)  1.2 (1.3)  0.2 (0.5)  0.9 (1.2)  0.1 (0.6)  0.5 (0.8) | 2.4 (1.7)  1.5 (1.6)  0.2 (0.6)  1.1 (1.5)  0.1 (0.7)  0.4 (0.7) | 3.3 (1.7)  1.7 (1.9)  0.6 (0.8)  0.9 (1.6)  0.2 (1.1)  0.3 (0.6) | 3.3 (1.7)  2.4 (1.5)  1.8 (1.4)  0.4 (0.8)  0.2 (0.7)  0.3 (0.7) |
| Total dairy | cup eq/1000 kcal | 0.7 (0.5) | 1.1 (0.6) | 1.4 (0.7) | 1.3 (0.9) | 1.2 (0.7) |
| Oils | g/1000 kcal | 14.2 (8.2) | 12.2 (6.5) | 9.9 (6.5) | 9.9 (6.0) | 9.5 (5.8) |
| Solid fats | g/1000 kcal | 13.5 (7.5) | 15.0 (7.3) | 15.5 (7.3) | 16.8 (7.7) | 17.1 (7.6) |
| Added sugars | tsp eq/1000 kcal | 18.8 (14.0) | 17.2 (11.9) | 16.0 (11.6) | 16.2 (13.8) | 14.6 (11.0) |

^a^ Vegetable totals do not include legumes.

^b^ The total protein foods group is a sum of animal and plant protein foods.

^c^ The meat group includes beef, veal, other ruminant animals, pork, and game.

^d^ The plant protein foods group includes all legumes, soybeans, nuts and seeds.
