# Supplementary material for: A Cross-Sectional Study of the Dietary Carbon Footprints of US Schoolchildren
Source: Nutrients. 2026 May 12;18(10):1529. doi: 10.3390/nu18101529 (PMC13209416; doi:10.3390/nu18101529)
Supplement: Supplementary file 1 [file nutrients-18-01529-s001.zip › Supplementary Table S5.docx]

**Supplementary Table S5.** Healthy Eating Index (HEI-2010) Component and Total Scores Across Greenhouse Gas Emission (GHGE) Quintiles: Findings from the 2014-2015 US School Nutrition and Meal Cost Study (SNMCS).

| **HEI component** | **Maximum score** | **Quintile 1**  **Low Greenhouse Gas Emission diet**  **n=433**  **Mean (SD)** | **Quintile 2**  **n=433**  **Mean (SD)** | **Quintile 3**  **n=433**  **Mean (SD)** | **Quintile 4**  **n=433**  **Mean (SD)** | **Quintile 5**  **High Greenhouse Gas Emission diet**  **n=433**  **Mean (SD)** |
| --- | --- | --- | --- | --- | --- | --- |
| Total fruit | 5 | 3.0 (2.0) | 3.1 (2.0) | 3.1 (1.9) | 3.1 (1.9) | 2.9 (2.0) |
| Whole fruit | 5 | 3.0 (2.2) | 3.0 (2.2) | 2.9 (2.2) | 2.9 (2.2) | 2.7 (2.2) |
| Total vegetables | 5 | 2.1 (1.6) | 2.0 (1.6) | 2.4 (1.6) | 2.4 (1.6) | 2.5 (1.6) |
| Greens and beans | 5 | 0.6 (1.6) | 0.5 (1.4) | 0.5 (1.4) | 0.5 (1.4) | 0.6 (1.5) |
| Whole grains | 10 | 4.8 (3.9) | 4.9 (3.7) | 4.5 (3.7) | 4.5 (3.7) | 4.3 (3.7) |
| Dairy | 10 | 5.3 (3.4) | 7.4 (3.0) | 7.5 (3.0) | 7.5 (3.0) | 7.5 (3.0) |
| Total protein foods | 5 | 3.3 (1.7) | 3.6 (1.6) | 4.0 (1.3) | 4.0 (1.3) | 4.6 (0.7) |
| Seafood and plant proteins | 5 | 0.8 (1.8) | 0.8 (1.7) | 0.8 (1.7) | 0.8 (1.7) | 1.1 (1.9) |
| Fatty Acids | 10 | 6.6 (3.6) | 5.3 (3.6) | 4.1 (3.4) | 4.1 (3.4) | 3.7 (3.2) |
| Refined grains ^a^ | 10 | 4.7 (3.7) | 5.1 (3.8) | 6.1 (3.6) | 6.1 (3.6) | 6.3 (3.5) |
| Sodium ^a^ | 10 | 5.9 (3.3) | 5.5 (3.2) | 4.2 (3.3) | 4.2 (3.3) | 3.6 (3.2) |
| Empty calories ^a,b^ | 20 | 17.3 (4.2) | 17.9 (3.5) | 17.6 (3.8) | 17.6 (3.8) | 18.0 (3.3) |
| Total HEI score | 100 | 57.4 (13.0) | 58.9 (12.8) | 57.8 (12.1) | 57.8 (12.1) | 57.7 (11.0) |

^a^ Higher component scores are considered beneficial. Thus, for refined grains, sodium, and empty calories, higher scores indicate diets that contain less of these items.

^b^ Calories from solid fats and added sugars
