# Supplementary material for: A Cross-Sectional Study of the Dietary Carbon Footprints of US Schoolchildren
Source: Nutrients. 2026 May 12;18(10):1529. doi: 10.3390/nu18101529 (PMC13209416; doi:10.3390/nu18101529)
Supplement: Supplementary file 1 [file nutrients-18-01529-s001.zip › Supplementary Table S6.docx]

**Supplementary Table S6.** Adjusted Healthy Eating Index (HEI-2010) Component and Total Scores in Low- and High–Greenhouse Gas Emission (GHGE) Diets: Findings from the 2014–2015 US School Nutrition and Meal Cost Study (SNMCS).

| **HEI component** | **Maximum score** | **Low Greenhouse Gas Emission diet**  **n=433**  **Adj. Mean^a^ (SE)** | **High Greenhouse Gas Emission diet**  **n=433**  **Adj. Mean^a^ (SE)** | **p-value^d^** |
| --- | --- | --- | --- | --- |
| Total fruit | 5 | 3.2 (0.1) | 3.0 (0.1) | 0.19 |
| Whole fruit | 5 | 3.2 (0.1) | 2.9 (0.1) | 0.03 |
| Total vegetables | 5 | 2.1 (0.1) | 2.5 (0.1) | <.0001 |
| Greens and beans | 5 | 0.6 (0.1) | 0.6 (0.1) | 0.86 |
| Whole grains | 10 | 5.1 (0.2) | 4.4 (0.2) | 0.008 |
| Dairy | 10 | 5.3 (0.2) | 7.4 (0.2) | <.0001 |
| Total protein foods | 5 | 3.3 (0.1) | 4.6 (0.1) | <.0001 |
| Seafood and plant proteins | 5 | 0.9 (0.1) | 1.2 (0.1) | 0.11 |
| Fatty Acids | 10 | 6.4 (0.2) | 3.7 (0.2) | <.0001 |
| Refined grains^b^ | 10 | 4.8 (0.2) | 6.4 (0.2) | <.0001 |
| Sodium^b^ | 10 | 5.9 (0.2) | 3.7 (0.2) | <.0001 |
| Empty calories^b,c^ | 20 | 17.4 (0.2) | 18.2 (0.2) | 0.003 |
| Total HEI score | 100 | 58.3 (0.7) | 58.4 (11.0) | 0.88 |

^a^ Adjusted for grade, race/ethnicity, gender, poverty level, and school meal consumption on the day of the dietary recall.
^b^ Higher component scores are considered beneficial. Thus, for refined grains, sodium, and empty calories, higher scores indicate diets that contain less of these items.
^c^ Calories from solid fat and added sugars.
^d^ Differences in mean values between low- and high-GHGE groups were assessed using independent samples t-tests.
